# Value of anatomical landmarks in single-nostril endonasal transnasal-sphenoidal surgery

**DOI:** 10.3892/etm.2013.936

**Published:** 2013-01-30

**Authors:** LIANG-FENG WEI, JINCHAO ZHANG, HONG-JIE CHEN, RUMI WANG

**Affiliations:** Department of Neurosurgery, Fuzhou General Hospital, Fujian Medical University, Fuzhou, Fujian 350025, P.R. China

**Keywords:** transsphenoidal approach, nasal cavity, sphenoid sinus, anatomical, landmark, pituitary

## Abstract

The sphenoid sinus occupies a central location in transsphenoidal surgery (TSS). It is important to identify relevant anatomical landmarks to enter the sphenoid sinus and sellar region properly. The aim of this study was to identify anatomical landmarks and their value in single-nostril endonasal TSS. A retrospective study was performed to review 148 cases of single-nostril endonasal TSS for pituitary lesions. The structure of the nasal cavities and sphenoid sinus, the position of apertures of the sphenoid sinus and relevant arteries and the morphological characteristics of the anterior wall of the sphenoid sinus and sellar floor were observed and recorded. The important anatomical landmarks included the mucosal aperture of the sphenoid sinus, a blunt longitudinal prominence on the posterior nasal septum, the osseocartilaginous junction of the nasal septum, the ‘bow sign’ of the anterior wall of the sphenoid sinus, the osseous aperture and its relationship with the nutrient arteries, the bulge of the sellar floor and the carotid protuberance. These landmarks outlined a clear route to the sella turcica with an optimal view and lesser tissue damage. Although morphological variation may exist, the position of these landmarks was generally consistent. Locating the sphenoid sinus aperture is the gold standard to direct the surgical route of TSS. The ‘bow sign’ and the sellar bulge are critical landmarks for accurate entry into the sphenoid sinus and sella fossa, respectively.

## Introduction

The pituitary gland is located below the center of the brain and over the sella on the cerebral surface of the body of the sphenoid. The sphenoid contains two sinuses, which open into the roof of the nasal cavity via the apertures on the posterior wall of the sphenoethmoidal recess directly above the turbinates. Since only thin layers of bone separate the sphenoid sinuses from the nasal cavities below and the sella turcica above, transsphenoidal surgery (TSS) is the first choice option for the removal of pituitary lesions rather than the transcranial approach.

The transsphenoidal approach has evolved considerably since it was first successfully performed by Schloffer in 1907 (1). Since then, TSS has been performed on numerous patients via different methods and the surgical routes are well formulated. However, even with the aid of fluoroscopy, the development of this technique was hampered by poor illumination and visualization of the surgical field. In 1967, Hardy first introduced the operating microscope to TSS, which laid a cornerstone foundation for the development of modern TSS (2). Since then, minimally invasive transsphenoidal surgical approaches to the sella turcica have undergone significant changes from sublabial transseptal, transnasal, to pure endonasal approaches. TSS has now become the standard approach for the surgical removal of pituitary adenoma (3,4). Compared with the transcranial approach, TSS does not require skin incision and external craniotomy, thereby offering the advantages of fewer complications, less discomfort and quicker recovery (5). Despite the increasing popularity of endoscopic techniques in recent years, microscopic TSS remains the mainstay of surgical treatment for pituitary lesions as it offers stereoscopic vision of the sella, excellent coaxial illumination and the capability for neurosurgeons to use traditional neurosurgical instruments (6). Moreover, access may be somewhat narrower in the absence of a nasal speculum, with some likening the endoscopic technique to ‘operating with chopsticks’ (7).

However, the surgical path of TSS is extremely deep and narrow, and the view is usually blocked by crucial neurovascular structures. In addition, the close proximity of the sphenoid sinus to the carotid artery and the optic canal, plus the high levels of variation between the anatomical structures of the sphenoid sinus and sellar floor, make the approach even more difficult, hence the success of the treatment greatly relies on the experience of the surgeon and the familiarity with anatomical landmarks through the surgical route. To date, the methods and techniques of TSS adopted by different surgeons with respect to surgical guidance and important landmarks vary significantly. Our knowledge regarding the anatomical structures relevant to TSS is mainly based on postmortem or imaging studies (8–13). However, the actual view at the surgical level under the microscope is different in real-world scenarios. However, in some patients with complex sellar anatomy, non-pneumatized sphenoid sinuses, or those undergoing reoperation, the typical appearance of the sella turcica and its relationship with the tuberculum sellae and clivus may be less conspicuous and identification of the midline is often more challenging, substantially increasing the risk of the surgery (14). Although advances in intraoperative neuronavigation have improved the accuracy associated with transsphenoidal and related extended endonasal skull base surgery over the last decade, they by no means obviate the requirement for knowledge of the relevant surgical anatomy. A practical anatomical study of the landmarks relevant to TSS is therefore warranted. We conducted a study based on 148 cases of endonasal TSS to delineate the important anatomical landmarks relevant to the three major regions across the procedure. We believe that these landmarks will provide useful guidance in clinical practice.

## Materials and methods

### General data

This study retrospectively reviewed 148 surgical records of single-nostril endonasal TSS for sellar lesions performed in our department in the period between May 2002 and February 2008. The patients included 78 males and 70 females with a mean age of 39.2 years (range, 12–78 years). Preoperative magnetic resonance imaging (MRI) was performed for all patients to assess variations of the sphenoid bone, sphenoid sinus and sellar floor. Postoperative histopathological examination confirmed the diagnosis of pituitary lesions. The study was approved by the ethics committee of Fuzhou General Hospital (Fuzhou, China). Written informed consent was obtained from all participants.

### Surgical procedure

**Figure 1:**
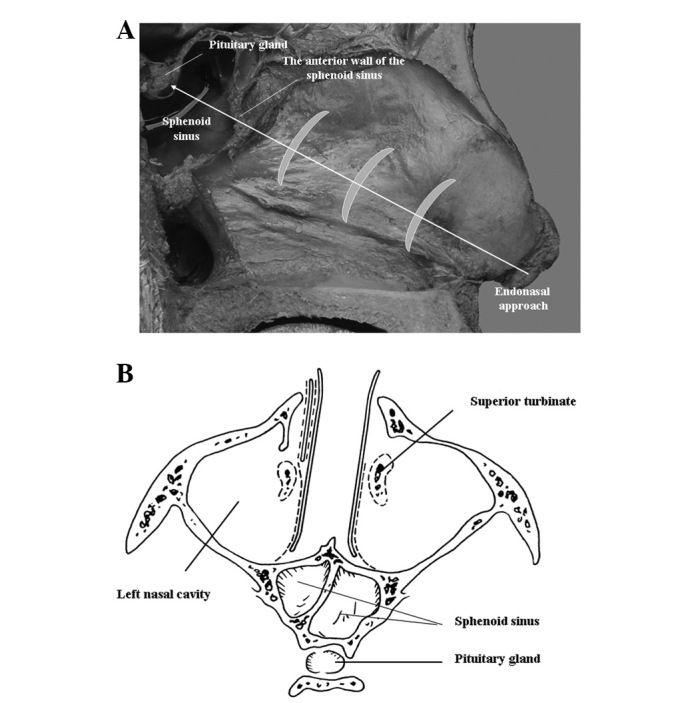
(A) Sagittal view of the pituitary gland, sphenoid sinus, and incisions of the right nasal septum. The white arrow indicates the direction of the endonasal transsphenoidal approach and the sickle-shaped gray figures indicate the position of the incisions on the nasal septum with methods A, B and C. (B) Axis view of the nasal cavity, sphenoid sinus and pituitary gland. Dotted line demonstrates the position of the mucoperiosteum on the nasal septum.

Patients lay in the supine position with the head extended by 20°. Surgeons were positioned directly behind the patient’s head. The microscope was orientated perpendicularly to the surface of the surgical floor first and then later adjusted towards the mucosal aperture of the sphenoid sinus. All surgeries were performed via a unilateral endonasal transsphenoidal approach (Fig. 1). This route approached the roof of the nasal cavity and the anterior wall of the sphenoid sinus and then entered the sphenoid sinuses by anterior sphenoidotomy followed by entry through the top of the sphenoid bone into the sella turcica. The method was modified three times with different positions of mucosal incision to obtain the optimal surgical view.

### Method A

An endoscope was used in 26 patients to examine and identify the nasal structures and the mucosal aperture of the sphenoid sinus. Under an operating microscope, the sphenoid sinus was approached either by expanding the aperture or by incising the ipsilateral mucoperiosteum at the posterior third of the nasal septum, fracturing the vomer and separating the bilateral mucoperiosteum to finally expose the anterior wall of the sphenoid sinus, followed by an anterior sphenoidotomy. The sphenoid septum was then excised, the orientation of the sellar floor was determined and the bony sellar floor and dura were opened to approach the pituitary gland and lesion. After removing the pituitary lesion, the dural defect of the sellar floor was closed with a small piece of autologous muscle harvested from the thigh and coated with fibrin glue. In a few difficult cases, neuronavigation was employed to guide access to the sella turcica.

### Method B

In another 63 patients, the surgical procedure was similar to method A. However, the mucoperiosteal incision was made on the posterior nasal septum (∼0.5–1.5 cm from the anterior wall of the sphenoid sinus) and then the perpendicular plate of the ethmoid bone was fractured and pushed to the opposite side before performing an anterior sphenoidotomy.

### Method C

In the final 59 patients, the mucoperiosteal incision was made at the osseocartilaginous junction of the nasal septum (∼3 cm from the naris). The cartilaginous nasal septum was pushed to the opposite side and the perpendicular plate of the ethmoid bone was excised to expose the anterior wall of the sphenoid sinus, followed by an anterior sphenoidotomy. The rest of the procedure was identical to the previous two methods.

### Anatomical assessment

The structure of the nasal cavity and sphenoid sinus, position of the apertures of the sphenoid sinus and relevant arteries and the morphological characteristics of the anterior wall of the sphenoid sinus and sellar bulge were observed and recorded. Nasal structures and anatomical anomalies that would affect the surgical approach were photographed and recorded.

## Results

### General outcomes

Comparing the three surgical methods, the approach with the mucoperiosteal incision made at the osseocartilaginous junction of the nasal septum provided a greater surgical view compared with the other two methods. The most common pituitary lesion was pituitary macroadenoma, occurring in 70.9% of patients (Table I). There were two cases of meningitis but no optic nerve or carotid artery injuries. The procedure-related complications included cerebrospinal fluid (CSF) leak (3.4%), mild subarachnoid hemorrhage (2.7%), nasal bleeding (6.8%) and mild nostril injuries (12.2%).

### Structure and anatomical landmarks located in the nasal cavities

**Figure 2:**
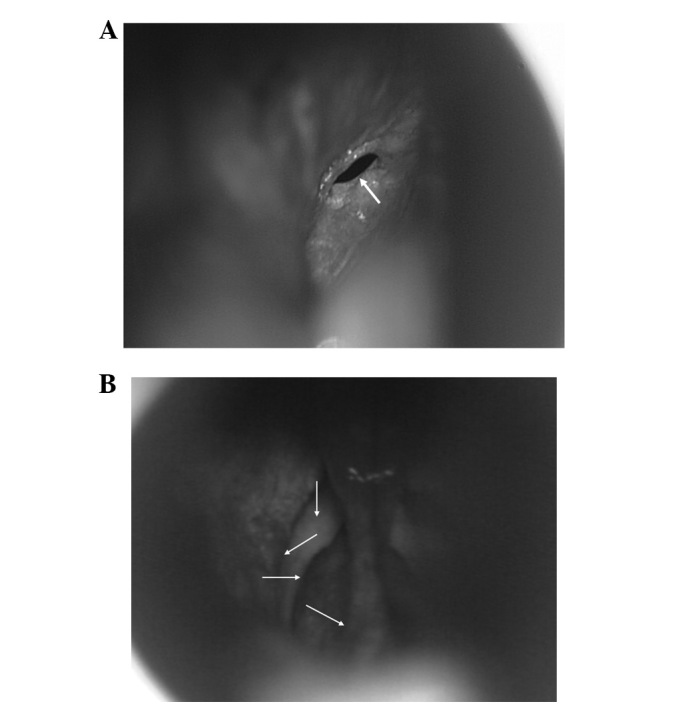
Structure of the right nasal cavity. (A) The mucosal aperture of the sphenoid sinus (arrow) observed after pushing the middle and superior turbinates laterally in the right nasal cavity. (B) The blunt longitudinal bulges of the posterior nasal septum (↙), anterior wall of sphenoid sinus (↓), superior turbinate (→), and middle turbinate (↘) as observed under the microscope.

The most important landmark in the nasal cavity is the mucosal aperture of the sphenoid sinus, which could be observed under the microscope in 79 patients (53.4%) after pushing the middle turbinate laterally (Fig. 2A). However, in the other 69 patients (46.6%), fracturing of the middle and superior turbinates was required. In addition, there was usually a blunt longitudinal bulge on the posterior nasal septum towards the sphenoid crest. The mucosal aperture was observed lateral to the end of this bulge (Fig. 2B). Once the aperture position was confirmed, the objective mirror of the microscope was fixed towards it. The sphenoid sinus together with the anteroinferior wall of the sella could then be easily approached from this direction.

### Anatomical landmarks after dissection of the nasal septal mucoperiosteum

**Figure 3:**
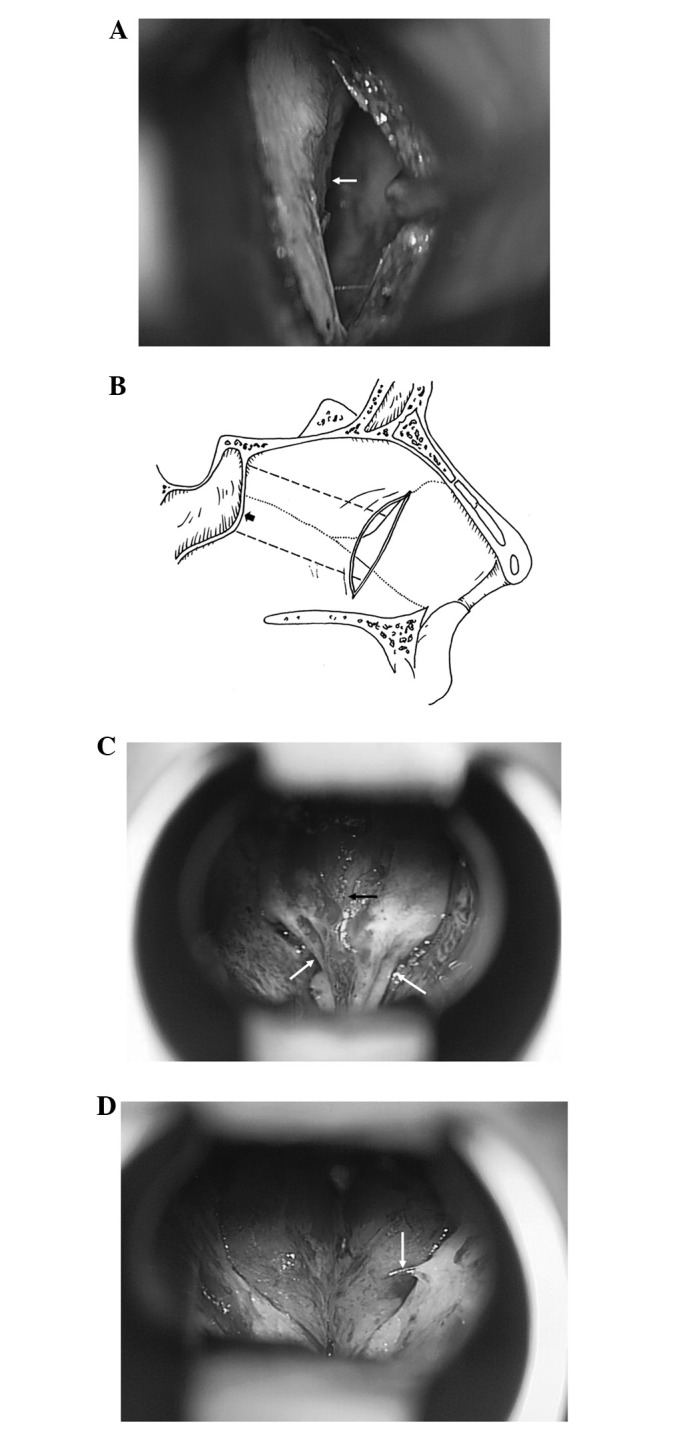
View after dissection of the septal mucoperiosteum exposing the anterior wall of the sphenoid sinus. (A) The bony and cartilaginous junction of the nasal septum (arrow) after incising the mucoperiosteum. (B) Sagittal view of the sub-mucoperiosteum approach. Dotted line indicates the direction of mucoperiosteum dissection. The arrow indicates the ‘shallowest point’ of the anterior wall of the sphenoid sinus. (C) The ‘bow sign’ of the anterior wall of the sphenoid sinus after excising the perpendicular plate of the ethmoid bone and the vomer. The medial anterior wall of the sphenoid sinus bulges anteriorly similar to a bird’s beak (black arrow) and bilateral bony apertures of the sphenoid sinus (white arrows) super laterally. (D) One artery (arrow) from the superior branch of the posterior septal artery was observed below the bony aperture.

In this region, the first landmark noted was the osseocartilaginous junction of the nasal septum, which could be observed after the septal mucoperiosteum was opened 3 cm from the naris (Fig. 3A). As illustrated (Fig. 3B), the anterior wall of the sphenoid sinus was situated at the end of the osseous septum, approximately perpendicular to the surgical view with the superior aspect tilting slightly posteriorly and the midline (the sphenoid crest) protruding anteriorly. This resembled a protruding bow under the microscope. The top of the bow was the perpendicular plate of the ethmoid bone, which was narrow and deeply tilted. The bottom of the bow was broad and shallow, bulged at the midline like a bird beak and two osseous apertures were located on both sides of the bulge (Fig. 3C). The ‘bow sign’ of the sphenoid bone is the most important landmark in this region, which indicated the correct direction of the approach. If the surgical route was directed slightly above the bulge, it would enter the ethmoid sinus. If the surgical route was directed below the bulge, it would not approach the sellar floor appropriately. The position of the ‘bird beak’ between the two apertures is the best place to enter the sphenoid sinus. In addition, there were usually 1–3 small nutrient arteries, 0.1–0.2 mm in diameter, arising from the superior branch of the posterior septal artery on each side. These arteries traveled inferomedially along the subperiosteum into the sphenoid bone below the osseous aperture of the sphenoid sinus. We found that one of these arteries consistently appeared at 3–7 mm inferior to the osseous aperture, which could serve as a surrogate marker for locating the aperture (Fig. 3D). Caution should be taken when separating the mucoperiosteum in this area in order to avoid damaging the blood vessels.

### Anatomical landmarks within the sphenoid sinus

**Figure 4:**
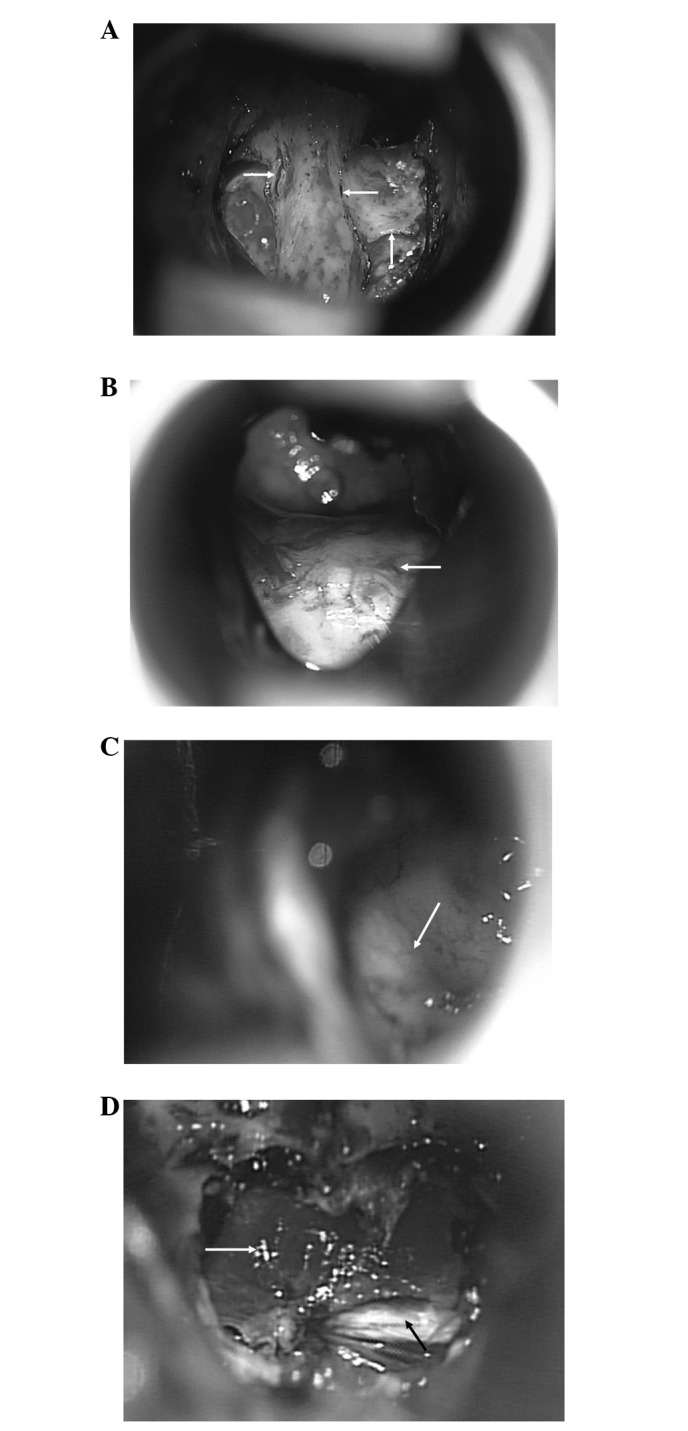
View inside the sphenoid sinus. (A) Complex septa and ridges inside the sphenoid sinus (arrows). (B) Bulging of the sellar floor observed after excising the septa of the sphenoid sinus. (C) When tilting the microscope slightly to the left, the protuberance of the internal carotid artery (arrow) could be observed on the left side of the saddle, corresponding to the cavernous segment of the carotid artery (arrow). (D) Large, orange-colored, thin pituitary gland (white arrow) beneath the sellar diaphragm could be observed after a pituitary microadenoma resection. The white dura (black arrow) could be observed on the surface of dorsum sella through the torn anterior sellar diaphragm.

The position, thickness, deviation and degree of development of the sphenoid septum varied greatly, which should be identified with the aid of preoperative MRI. In our study, most patients had only one sphenoid septum but multiple sphenoid septa were observed in a few cases (Fig. 4A). The important landmark in the sphenoid sinus is the bulge of the sellar floor (Fig. 4B), which is beneath the pituitary fossa. Its morphology varied in accordance with the degree of the sphenoid sinus pneumatization and the development of the pituitary fossa. It may also present as an increased inferior and lateral convexity or destruction in patients with pituitary macroadenomas. We were able to define this bulge in most patients. When this bulge was ill-defined, the observation of carotid prominence would be useful as it was always located in the parasellar space and could be observed after tilting the microscope slightly to the left or right (Fig. 4C). In the present study, successful access to the sella fossa was achieved in all our patients. Neuronavigation was only used in a few difficult cases during the early periods of our practice.

### Differentiation of the pituitary gland and dura

The outcome of TSS is related to the proper removal of lesions and the protection of normal pituitary gland tissue. As shown in Fig. 4D, normal pituitary gland tissue was orange-colored and tough, whereas pituitary nervous tissue was pale and soft. It is also important to distinguish the type of dura to confirm whether the surgical approach is appropriate or not. The differences in appearance and texture between the dura of the anterior wall of the pituitary turcica and that of the tuberculum sella could be identified. The former was smooth and thin without any pattern, usually orange, light blue, light yellow or white in color. The latter was white and thick with a horizontal streaky pattern and larger collagen fiber bundles, which were located beneath the anterior lamina terminalis cistern, inter-hemispheric cistern and gyrus rectus.

## Discussion

In the present study, we delineated important anatomical landmarks for endonasal TSS, including the mucosal aperture of the sphenoid sinus, a blunt longitudinal prominence on the posterior nasal septum, the osseocartilaginous junction of the nasal septum, the ‘bow sign’ of the anterior wall of the sphenoid sinus, the osseous aperture and its relationship with nutrient arteries, the bulge of the sellar floor and the carotid protuberance. These landmarks outline a clear route to the sella turcica providing the optimal view and causing less tissue damage. Based on these landmarks, we successfully accessed the sella turcica and dissected pituitary lesions in all patients without any assistance from intraoperative CT scan and fluoroscopic navigation.

Several postmortem and imaging studies attempting to illustrate anatomical landmarks for TSS have been conducted previously (6). Using cadaveric heads and 10 skulls, Campero *et al* produced a spheno-sellar point and a spheno-nostril line to guide the head positioning for TSS (8). However, the clinical applicability of such types of measurement is limited due to the small number of subjects and the difficultly in evaluating the procedure as a result of limited standard verification, despite being attempted in 102 surgical procedures. Other studies have tried to disclose the variations of human skulls which may affect the transsphenoidal approach. Campero *et al* studied dry skulls and found that the location of apertures varied greatly (9). Tatreau *et al* (15) reported that the periform aperture and pneumatization to the planum and sella changed with age in pediatric patients, but not in adult patients. Hamid *et al* (10) used CT and MRI scans to study variations of the sphenoid sinus in 296 patients with pituitary lesions. The authors found that the degree of pneumatization of the sphenoid sinus varied greatly but the appearance of the sellar bulge was prominent, which appeared in 75% of patients. In the present study, we did not find a great variation in the position of aperture. If this is the case, we may still use other landmarks, such as the blunt longitudinal prominence on the posterior nasal septum and the nutrient arteries, to determine its position to guide surgical direction. The appearance of these landmarks, especially the position of nutrient arteries, was prominent. We also identified the sellar bulge in most patients, which is consistent with the study by Hamid *et al* (10). In the cases of ill-defined sellar bulges, the identification of the carotid protuberance will be useful to decide the position of the sella. Within all these anatomical landmarks, the ‘bow sign’ on the anterior wall of the sphenoid sinus is the most important and has thus far never been reported in previous studies. The ‘bow sign’ was consistent in the majority of patients, thus it is useful to guide the surgical direction towards the sella turcica regardless of the variation in the location of apertures. The combination of all these landmarks can therefore minimize the risk of anatomic disorientation so as to avoid major complications in our patients. In addition, we made the mucoperiosteal incision in three distinct positions of the nasal septum and found that the approach from the incision at the osseocartilaginous junction provided the optimal surgical view and direction. This incision was also recommended by Marquardt *et al* (16).

Over the last 10 years, endoscopic techniques have seen a marked development and led to a trend in transsphenoidal surgical approaches. However, the preference of endoscopic TSS or microscopic TSS depends on the technological refinements and economical restraints. Although an endoscopic approach permits a better view in the sphenoid sinus and the parasellar region (17), it cannot magnify the area being viewed and solve the narrowness of the anatomical spaces and the consequent limited view before sphenoidotomy, which is an advantage of the microscopic approach (16). It also has difficulty in intracranial hemorrhage control and the closure of the dural and osseous defects following tumor dissection, subsequently increasing the risk of postoperative CSF leak, meningitis, etc. (18). Goudakos *et al* (19) reviewed all studies from 1952 to 2010 regarding endoscopic TSS and microscopic TSS and demonstrated that the two techniques provided similar rates of complete tumor excision and remission rates. Endoscopic surgery was associated with fewer complications related to surgical technique. However, another study identified no statistically significant differences between the two approaches (20). It should be noted that these studies did not take the surgical experience or concomitant use of intraoperative imaging modalities into account. Nevertheless, we did not underestimate the value of the endoscopic approach even though most surgeries were performed under the microscope. The endoscope was used to aid the establishment of surgical techniques in the first 26 patients of the present study. In fact, the endoscopic approach for pituitary lesions involves exactly the same surgical steps as the microscopic approach. As described by Jho *et al* (21,22), it opens exactly the same anatomical tissue layers and provides an identical wide exposure of the concerned structures as a microscopic approach. Therefore, the anatomical landmarks that were summarized from our experience with microscopic TSS can be also applied to endoscopic approaches.

Although the aforementioned anatomical landmarks are useful to guide the surgical procedure, neuronavigation techniques are invaluable in evaluating the variation of sphenoid sinus and the sellar region, especially in those with residual or recurrent masses in the setting of previous TSS, which may inevitably alter the normal anatomical structure of the skull base (23). In the present study, we used neuronavigation to distinguish the sellar structure in the few patients we experienced difficulties in identifying the sellar floor in the early period of the present study. Therefore, neuronavigation techniques and anatomical landmarks are complementary to each other. The combination of the two may improve surgical outcomes.

Since we mainly focused on endonasal TSS, we did not compare our findings with those using another surgical approach. We believe, however, that the anatomical landmarks for endonasal TSS are also applicable to other approaches.

Locating the sphenoid sinus aperture is the gold standard to direct the surgical route of TSS. The ‘bow sign’ and the sellar bulge are critical landmarks for the accurate entry into the sphenoid sinus and sella fossa. These landmarks outline a clear route to the sella turcica with the optimal view causing less tissue damage. The application of these landmarks will aid the reduction of complications and improvement of outcomes of TSS.

## Figures and Tables

**Table I:** Diagnosis of pituitary lesions.

Pituitary lesions	Number of cases
Pituitary adenoma	136
Knosp-Steiner classification for parasellar extension	
0–II	122
III	24
IV	2
Classification by size	
Microadenoma (≤10 mm)	10
Macroadenoma (10–40 mm)	105
Giant adenoma (≥40 mm)	21
Dural invasion on the sellar floor	13
Rathke cysts	4
Sphenoid mucocele	2
Pituitary abscess	2
Empty sella	2
Meningioma	1
Metastatic tumor	1
Pituitary hyperplasia	1
